# Assessing motor deficits in compressive neuropathy using quantitative electromyography

**DOI:** 10.1186/1743-0003-7-39

**Published:** 2010-08-11

**Authors:** Joseph Nashed, Andrew Hamilton-Wright, Daniel W Stashuk, Matthew Faris, Linda McLean

**Affiliations:** 1 School of Rehabilitation Therapy, Queen's University, Kingston, Ontario, Canada; 2 Department of Systems Design Engineering, University of Waterloo, Waterloo, Ontario, Canada; 3 Physical Medicine and Rehabilitation, Queen's University, Kingston, Ontario, Canada

## Abstract

**Background:**

Studying the changes that occur in motor unit potential trains (MUPTs) may provide insight into the extent of motor unit loss and neural re-organization resulting from nerve compression injury. The purpose of this study was to determine the feasibility of using decomposition-based quantitative electromyography (DQEMG) to study the pathophysiological changes associated with compression neuropathy.

**Methods:**

The model used to examine compression neuropathy was carpal tunnel syndrome (CTS) due to its high prevalence and ease of diagnosis. Surface and concentric needle electromyography data were acquired simultaneously from the abductor pollicis brevis muscle in six individuals with severe CTS, eight individuals with mild CTS and nine healthy control subjects. DQEMG was used to detect intramuscular MUPTs during constant-intensity contractions and to estimate parameters associated with the surface- and needle-detected motor unit potentials (SMUPs and MUPs, respectively). MUP morphology and stability, SMUP morphology and motor unit number estimates (MUNEs) were compared among the groups using Kruskal-Wallis tests.

**Results:**

The severe CTS group had larger amplitude and longer duration MUPs and smaller MUNEs than the mild CTS and control groups, suggesting that the individuals with severe CTS had motor unit loss with subsequent collateral reinnervation, and that DQEMG using a constant-intensity protocol was sensitive to these changes. SMUP morphology and MUP complexity and stability did not significantly differ among the groups.

**Conclusions:**

These results provide evidence that MUP amplitude parameters and MUNEs obtained using DQEMG, may be a valuable tool to investigate pathophysiological changes in muscles affected by compressive motor neuropathy to augment information obtained from nerve conduction studies. Although there were trends in many of these measures, in this study, MUP complexity and stability and SMUP parameters were, of limited value.

## Background

Compression neuropathies are extremely prevalent [1] and are associated with a wide array of sensory and motor deficits [2]. Nerve conduction studies are used to assess the integrity of motor and sensory nerves through estimates of nerve conduction velocity and response amplitudes [3,4]. Unfortunately these electrophysiological methods are limited since they do not directly measure the pathophysiological changes occurring within the motor unit pool [3,4]. For example, compound muscle action potential (CMAP) amplitude might be reduced both in cases of conduction block and in cases of demyelination [3,4]. Studying the changes that occur at the motor unit level in compressive neuropathies might be of considerable value in providing insight into the extent of motor unit loss and neural re-organization resulting from nerve compression injury. This approach may therefore significantly augment the information available from nerve conduction studies.

Quantitative electromyography (EMG) [5-7] may be used to provide information about the re-organization of motor units following nerve injury and/or muscle disease. One such approach, decomposition-based quantitative electromyography (DQEMG), has been shown to be a valid and reliable [8,9] method and has been used to assess changes in motor unit (MU) size, fibre density and firing rate, as well as differences in MU number estimates between healthy subjects and patients with neurologic or myopathic diseases [7,10-13]. The assessment of MU potential (MUP) morphology and stability, MU number estimates (MUNEs) and MU activation patterns may provide insight into the pathophysiological processes associated with peripheral nerve compression injuries; however quantitative EMG techniques have not been tested for such a purpose.

The purpose of this study was to determine the feasibility of using DQEMG to study motor pathology seen in compression neuropathy. Carpal tunnel syndrome (CTS) provides a convenient model of compression neuropathy for such an investigation since nerve conduction studies can be used to stratify subjects with and without motor nerve involvement. As such, this study was designed to compare quantitative EMG data among a group of subjects with severe CTS (i.e. those with signs of motor nerve involvement), a group with mild CTS (i.e. those with nerve compression but no evidence of motor nerve injury) and a group of healthy control subjects. In particular, we aimed to determine whether there was measureable evidence of collateral sprouting or motor axon loss in individuals with severe CTS as compared to those with mild or no CTS.

## Methods

### Participants

The study was approved by the Queen's University Health Sciences Research Ethics Board and all subjects provided informed consent prior to participation. Potential participants were recruited through advertisements and physician referral in the Kingston, Ontario (Canada) community. Volunteers between the ages of 18 - 60 [14]. Potential participants were screened to ensure that they had no previous injury to the neck or upper limbs, no medical diagnosis of neurological or metabolic conditions [15], and no signs or symptoms of cervical radiculopathy or inflammation of the joints of the neck or upper limb. Those who met these eligibility criteria underwent electrophysiological screening to determine whether they fit within one of three strata (no CTS, mild CTS or severe CTS). On arrival at the laboratory, potential participants underwent Spurling's compression and distraction tests [16]. If their symptoms of pain or paraesthaesias diminished or were exacerbated during or following the tests, that participant was excluded from the study. Subjects with CTS were required to have symptoms including hand paraesthesias and hypoesthesia or pain in the first three digits [2].

### Electrophysiological Examination

Subjects with CTS were included on the basis of a clinical and electrophysiological examination, which classified them as having either mild or severe CTS, and control subjects were required to have no evidence of sensory or motor nerve conduction abnormalities. Subjects with electrophysiological evidence of moderate CTS were excluded from the study since clear differentiation between subjects with sensory involvement only and those with both sensory and motor involvement was desired.

Nerve conduction studies were performed using the Comperio™ (Neuroscan Medical Systems, El Paso,Texas) Clinical EMG system. Palmar temperatures were monitored and maintained above 30°C for all testing. Prior to electrode placement, the hand under investigation was thoroughly cleaned using compound rubbing alcohol (Life™, Toronto, ON) and gauze pads. Surface EMG signals were detected using self-adhering electrocardiogram electrodes (Harris Healthcare, Hudson, MA) cut in half to measure 1 cm × 3 cm. A full-sized (2 cm × 3 cm) electrode was placed on the posterior aspect of the hand to serve as a reference. Signals were amplified (Neuroscan Medical Systems, El Paso, TX) with a bandpass filter of 5 Hz - 5 kHz, digitized and stored using the Comperio Software by Neuroscan.

Only the affected upper limb was tested in individuals with CTS. If both hands were symptomatic, the side with more severe symptoms was evaluated. All participants were required to have normal conduction velocity of both the median and ulnar nerves in the forearm. Subjects were then stratified by CTS severity using the following criteria:

Healthy: No nerve conduction study based evidence of sensory or motor impairment.

#### Mild CTS

prolongation of sensory distal latencies (median mid palmer latency > 2.2 ms or prolongation of the median mid-palmar CNAP relative to the ulnar mid-palmar CNAP > 0.4 ms or a difference in latency > 0.5 ms between median and ulnar SNAPs of digit four); [4,17].

#### Severe CTS

prolongation of both median motor (CMAP > 4.4 ms) and sensory distal latencies (median mid palmer latency > 2.2 ms or prolongation of the median mid-palmar CNAP relative to the ulnar mid-palmar CNAP > 0.4 ms or a difference in latency > 0.5 ms between median and ulnar SNAPs of digit four); with either an absent SNAP, or low amplitude thenar CMAP [4,17].

### Experimental Protocol

Demographic data were documented for each participant, including height, weight, age, occupation and handedness. Each participant completed a self-administered Carpal Tunnel Syndrome Questionnaire [18] to quantify the functional limitations associated with their condition, which was used for descriptive purposes.

EMG data were acquired using AcquireEMG™ software on the Neuroscan Comperio™ system (Neuroscan Medical Systems, El Paso, TX). Intramuscular signals were detected using disposable concentric needle electrodes (Model 740 38-45/N; Ambu Neuroline, Baltorpbakken, Ballerup, Denmark) and amplified with a bandpass of 10 Hz to 10 kHz. Surface signals were detected using self-adhering 1 cm × 3 cm electrocardiogram electrodes (Harris Healthcare, Hudson, MA) and amplified with a bandpass of 5 Hz to 1 kHz. A monopolar surface electrode configuration was used to record CMAPs and for data acquisition of SEMG data. The anode was placed over the belly of the APB muscle and the cathode was located over the APB tendon.

Subjects were first asked to perform an isometric maximum voluntary contraction (MVC) by pushing their thumb into the examiner's resistance for 10 s. The root mean square (RMS) value of the EMG signal over contiguous 1s intervals was calculated and the highest RMS value across the 10 s was determined to be the RMS value of the MVC (RMSMVC).

The concentric intramuscular electrode was then inserted into the APB such that the tip of the electrode was located within the muscle and beneath the surface electrode. Needle and surface EMG data were acquired simultaneously with sampling rates of 31,250 and 3125 Hz respectively. With the needle in situ, the subject was instructed to increase the level of isometric contraction of the APB until MUPs from several active motor units were detected. The needle position was then adjusted to ensure the detection of 'sharp' MUPs with short rise times, indicating that the needle tip was in close proximity to a sample of motor units. The amplitude of contractions was described as a percentage of the RMSMVC although participants were not instructed to contract at a given percentage of their MVC. Instead subjects were instructed to increase the contraction intensity until the aggregate number of MUPs detected per second, as estimated through the number of pulses per second (pps) was approximately 60 and to maintain this level of contraction as consistently as possible throughout a 30 s period of data acquisition. By standardizing the intensity of the contraction, participants were contracting their APB with similar numbers of active motor units. This is because in healthy or unhealthy APB muscles during low to moderate levels of activation motor unit firing rates across active APB motor units are similar (approx. 8 - 12 pps). At the end of the 30 second contraction, the subject was instructed to relax their muscle while the needle position was changed to detect MUPs from more superficial, intermediate, or deep portions of the muscle in an attempt to sample from a broad distribution of MUs. Data collection from submaximal contractions continued until at least 30 acceptable MUPs were detected, which required five to eight contractions from each subject. The acceptability criteria are discussed below.

DQEMG was used to decompose the needle-detected EMG data into MUPTs. For each MUPT a MUP template was calculated using median-trimmed averaging of the 51 most similar MUP samples from the train. The associated SMUP for each MUPT was estimated using spike triggered averaging of the surface-detected EMG signal, which used all of the occurrences within the MUPT over the 30 s data acquisition period [11]. To be included in the data set and therefore in subsequent analyses, a SMUP had to be temporally aligned (within 10 ms) with its corresponding MUP and verified as a distinct waveform with respect to the RMS of the signal baseline.

### Acceptability Criteria for MUPs and SMUPs

The EMG data from each 30 s contraction was decomposed immediately after the contraction was completed such that the number of acceptable MUPs could be monitored. As noted above, data collection continued until at least 30 acceptable MUPs were detected from each subject, which required between 5 and 8 contractions lasting 30 seconds each.

MUPTs were evaluated during off-line analysis. Two interrelated criteria were used to determine the acceptability of a given MUPT: the variability in the instantaneous firing rate versus time plot (generated in the DQEMG output), and the inter-discharge interval (IDI) histogram. An acceptable train had at least 51 MUPs used to create the template, a firing rate in the physiological range (8-30 Hz) with a coefficient of variation lower than 0.20, as well as an inter-discharge interval (IDI) histogram that was Gaussian-shaped and had a coefficient of variation lower than 0.30 [11]. Any MUPTs identified by DQEMG that did not meet all of these criteria were excluded from the analysis. Markers indicating the onset, negative peak, positive peak and end of the MUP waveforms, and markers indicating the onset, negative peak onset, negative peak, positive peak, and end of the SMUP waveforms were automatically determined by the DQEMG software, but were visually inspected for accuracy, and manually repositioned if incorrectly placed.

## Data Reduction and Analysis

### Motor Unit Potential Morphology

The MUP template parameters included in the analysis were peak-to-peak amplitude, duration, number of phases, number of turns and fibre count. Fibre count was calculated as the number of significant peaks in the acceleration filtered MUP template [7]. The SMUP parameters that were included in the analysis were peak-to-peak amplitude, duration and negative peak area.

### Motor Unit Potential Stability Measures

DQEMG algorithms for analyzing the variability of the MUPs within a MUPT were used to obtain measures of MUP stability [7]. Across the ensemble of isolated MUPs within a MUPT, acceleration filtering was used to measure acceleration variability or jiggle (Ajiggle) [7]. In addition, the standard deviation of the distances of the MUPs of a train to its MUP template divided by the mean of the distances of the MUPs of a train to its MUP, termed the shimmer coefficient of variation (shimmerCov), was calculated as a second measure of stability. Differences in shape were measured using the time domain samples of the MUPs and MUP template as features and the Euclidian distance metric [7].

### Motor Unit Number Estimates

Motor unit number estimates (MUNEs) [8] were calculated by dividing size related parameters of the maximum CMAP by the same size related parameter of the ensemble averaged or mean SMUP (mSMUP) calculated using the negative peak onset aligned SMUPs estimated for the muscle. Three different parameters were used to calculate MUNEs: peak-to-peak amplitude, negative peak amplitude and negative peak area.

### Statistical Analysis

All data analyses were performed using MINITAB^® ^Statistical Software (v.15). The MUP and SMUP data were averaged for each muscle studied to provide average MUP and SMUP parameter values for each participant. Due to the small sample size and non-normal distribution in many variables, non-parametric statistics were performed and as such, all measures are described and compared among groups using the median value and interquartile range (IQR). Between-group differences were assessed for all data (the questionnaire data, the MUP and SMUP parameter values and the MUNEs) using Kruskal-Wallis tests (alpha = 0.05). Post hoc analyses were performed using Mann-Whitney U tests.

## Results

### Subjects

Twenty eight volunteers passed the telephone screening and agreed to participate in the study. One volunteer was excluded after clinical evaluation screening because of suspected radiculopathy. Two volunteers were excluded after neurophysiological evaluation as they were classified as having moderate CTS. Two other volunteers were excluded due to the discovery that they had confounding conditions (pregnancy and rheumatoid arthritis, respectively). In the end, nine men and fourteen women participated in the study: 9 healthy individuals (4 men, 5 women), 8 individuals with mild CTS (2 men, 6 women) and 6 individuals with severe CTS (3 men, 3 women). There were no differences in the median age or sex among the groups (Table 1; p > 0.05). There were significant differences between the duration of symptoms of each group, however this was expected (Table1; p < 0.05).

**Table 1:** Demographic data.

Group	Sex	Age (Years)	Duration of Symptoms (Months)	Intensity (pps)	%MVC
**Control**	4 Men, 5 Women	43.0 (30.0-53.5)	0 (0-0)	12.71 (11.45-15.5)	10.04 (8.84-21.13)
**Mild CTS**	2 Men, 6 Women	46.0 (41.3-52.5)	5.5(2.3-7.5)	12.86 (11.58-14.95)	13.6 (8.06-21.39)
**Severe CTS**	3 Men, 3 Women	53.5 (41.3-57.8)	13 (7.0-19.0)	10.52 (1.23-12.56)	39.6 (31.95-44)

Medians and interquartile ranges are presented. * denotes a significant difference from parameters notated with**, pps = pulses per second; MVC = Maximum Voluntary Contraction

The intensity of the contractions, did not differ significantly among the groups (Table 1; p > 0.05). During EMG signal acquisition, in order to achieve adequate signal intensity (approximately 60 pps) the isometric contractions of the severe CTS group were performed at a significantly higher percentage of their MVC compared to the mild CTS and control groups (Table 1; p < 0.05). This 'late recruitment' (i.e. recruitment of motor units at higher levels of contraction) is in itself an indication of collateral reinnervation as the muscle adapts to motor unit loss.

As expected, since the groups were stratified based on these values, significant group differences were found for all CMAP characteristics (negative-peak amplitude; p < 0.05, peak-to-peak amplitude; p < 0.05 and negative-peak area; p < 0.05) as indicated in Table 2. Post hoc analysis revealed significant differences in these parameters between the healthy control group and both the mild (p < 0.05) and severe CTS (p < 0.05) groups for all three morphological features.

**Table 2:** CMAP morphology.

Group	Pk-Pk Amplitude (μV)	Neg Pk Amplitude (μV)	Neg Pk Area (μVms)
**Healthy**	19797 (17790-23458)*	11830 (10741-12922)*	31468 (29964-41797)*
**Mild CTS**	12940 (10447-14175)**	7518 (6824-8889)**	22114 (17452-28462)**
**Severe CTS**	10053 (8242-15437)**	6447 (4884-8311)**	21749 (15994-31206)**

Medians and interquartile ranges are presented.* denotes a significant difference from parameters notated with **, Neg Pk = negative-peak; Pk-Pk = peak-to-peak

### Symptom Severity and Functional Deficits

Data from the Boston Carpal Tunnel Questionnaire indicated that there were significant group differences in symptom severity scores (Severe CTS: 4.0 (IQR: 3.18-4.45), mild CTS: 3.09 (IQR: 2.91-4.00), control: 1.0 (IQR: 1.00-1.05); p < 0.05) and functionality scores (Severe CTS: 3.4 (IQR: 2.6-4.1), mild CTS: 1.2 (IQR: 1.0-2.1), control: 1.0 (IQR: 1.0-1.2); p < 0.05). Post hoc analysis revealed significant group differences in symptom severity between the healthy control group and both the mild (p < 0.05) and severe CTS groups (p < 0.05) and in functionality scores between the severe CTS group and the healthy control groups (p < 0.05).

### **MUP Morphology**

Significant group differences were found in the MUP amplitudes (p < 0.05) as identified in Table 3. The severe CTS group demonstrated larger peak-to-peak MUP amplitudes compared to the mild CTS and control groups. There was no difference in peak-to-peak MUP amplitude between the mild CTS and the control groups.

**Table 3:** Needle- and Surface-Detected MUP morphology measures.

		Needle-detected MUPs			Surface-detected MUPs		
	
Group	Pk-Pk Amplitude (μV)	Duration (ms)	No. of Turns	No. of Phases	Amplitude (mV)	Neg Pk Area (mVms)	Duration (ms)
**Control**	410.9 (299.8-490.2)†	6.8 (5.6-9.0)†	3.3 (2.9-3.8)	2.6 (2.3-2.8)	151.0 (123.0-172.0)	263.0 (226.4-321.0)	27.5 (23.5-30.7)
**Mild CTS**	482.9 (448.1-589.4)†	7.3 (6.4-9.8)†	3.3 (2.9 -3.7)	2.7 (2.2-2.8)	213.5 (104.3-289.3)	341.3 (162.4-467.5)	26.1 (22.2-29.4)
**Severe CTS**	690.9 (561.4-821.2)*	10.5 (8.2-12.6)*	3.8 (3.1-4.1)	3.0 (2.8-3.5)	284.0 (129.8-420.3)	519.0 (237.0-790.0)	34.2 (28.7-38.7)

Medians and interquartile ranges are presented. * denotes a significant difference from parameters notated with †, Pk-Pk= peak-to-peak; Neg Pk = negative peak

Similar to the MUP amplitude results, the severe CTS group demonstrated longer duration MUPs than both the control and mild CTS groups (Table 3; p < 0.05). No significant difference in duration was found between the mild CTS group and the control group (Table 3).

No group differences among the three groups were found in either the average number of phases or turns seen in the MUPs (Table 3). It is noteworthy, however, that trends indicating collateral sprouting were evident in that the severe CTS group tended to have more phases and turns in their MUPs. Similarly, the Ajiggle, shimmerCov and fibre count (Table 4) data did not demonstrate any significant differences among the three groups (p > 0.05), but did show trends whereby the amount of Ajiggle and ShimmerCov increased with severity of CTS.

**Table 4:** MUP stability measures.

Group	Fibre Count	Ajiggle	ShimmerCov
**Control**	1.5 (1.4-1.7)	0.17 (0.15-0.19)	0.53 (0.45-0.57)
**Mild CTS**	1.7 (1.6-2.1)	0.19 (0.17-0.22)	0.62 (0.53-0.67)
**Severe CTS**	1.7 (1.3-2.0)	0.20 (0.15-0.24)	0.63 (0.56-0.71)

Medians and interquartile ranges are presented

### SMUP Morphology

The Kruskal-Wallis tests failed to reveal any significant differences among the groups for any of the SMUP morphology parameters (amplitude, area, duration) as demonstrated in Table 3.

### MUNE

**Figure 1:**
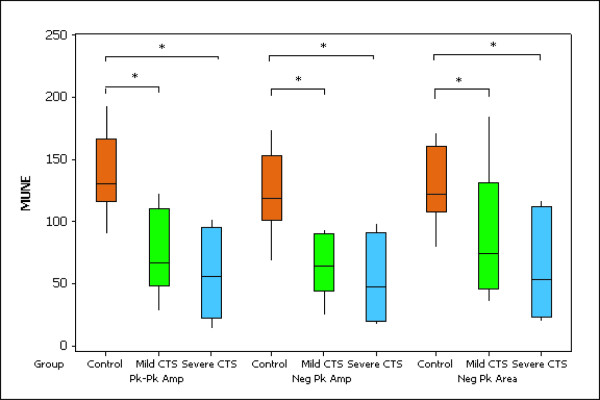
**Box plots of Abductor Pollicis Brevis MUNE values calculated using the spike triggered average technique**. Pk-Pk Amp = peak to peak amplitude, Neg Pk Amp = negative peak amplitude, Neg Pk Area = negative peak area. Mild = mild CTS group, Severe = severe CTS group. The boxes represent the interquratile range with the bar within each box representing the median value. The whiskers extend to the maximum and minimum data points within 1.5 box heights from the top and bottom of the box respectively (* denotes significant differences between groups)

The results of the MUNE calculations are summarized in Figure 1. Significant group differences were found for all three methods of calculating the MUNE, whereby significant group differences were found between the control group and both the mild and severe CTS groups (peak to peak amplitude; p < 0.05, negative peak amplitude; p < 0.05 and negative peak area; p < 0.05). No significant differences in MUNEs were found between the mild and severe CTS groups regardless of the method of calculation. (Figure 1).

## Discussion

The purpose of this study was to determine the feasibility of using DQEMG as a means of determining pathophysiological mechanisms associated with motor deficits in compressive neuropathy. A significant aspect of the EMG signal detection protocol was that the subjects were instructed to create constant-intensity as opposed to constant %MVC force contractions. At low to moderate levels of activation, where motor unit firing rates are similar, the constant-intensity protocol results in the activation of similar numbers of motor units across various sets of muscles. The constant-intensity protocol will therefore accentuate changes in motor unit recruitment. For myopathic muscles with fewer and smaller diameter fibres 'early recruitment' (i.e. recruitment of motor units at lower levels of contraction) during constant-intensity protocols will result in reduced %MVC contractions. In contrast, for neurogenic muscle with motor unit loss and collateral reinnervation 'late recruitment' during constant-intensity protocols will result in increased %MVC contractions. In both cases, eliciting the altered recruitment, which occurs to compensate for muscle changes, produces EMG signals that can be more effectively used to detect underlying muscle changes. Because %MVC force measurement is impossible for some muscles and clinically impractical for most while most clinical EMG machines now provide an intensity measure, constant-intensity protocols (albeit at lower levels of intensity than used in this study) are used during clinical needle EMG examinations. In this study, 'late recruitment' resulted in significant changes in the levels of %MVC at which the EMG data was detected for the severe CTS group relative to the mild CTS and healthy groups. In addition, MUP morphology data revealed that individuals with severe CTS had larger amplitude and longer duration MUPs than the other two groups. Both of these differences are consistent with motor unit loss, collateral sprouting and assimilation of orphaned muscle fibers. No differences were seen in SMUP morphology or MUP complexity and stability between the groups. It is not clear whether MUP complexity and stability measures were not sensitive enough to detect differences between the groups, or whether there truly were no differences in MUP complexity and stability between the groups. Both the CMAPs and MUNEs suggested that individuals with severe CTS, who were selected based on evidence of motor deficits obtained from nerve conduction studies, and those with mild CTS who had no nerve conduction study based evidence of motor conduction block or delay (since their CMAPs were within normal limits), had evidence of axonal loss relative to the control subjects. These results indicate that the use of a constant-intensity protocol and DQEMG may provide useful information in the assessment of MUP morphological changes associated with compressive neuropathies and may augment information available from nerve conduction studies. In particular, constant-intensity based use of DQEMG, by virtue of its ability to detect differences in MUP morphology may be useful in determining whether a muscle adapts to a compressive neuropathy by using collateral sprouting as compared to axonal regeneration.

### Participants

Subject recruitment for this study proved to be very difficult despite the high prevalence estimates for CTS [1]. Recruitment was limited particularly by the exclusion criteria that required individuals to be between the ages of 18-60 and to have no other pain complaints or potentially confounding pathology, as well as our decision to target individuals with mild or severe CTS but not moderate CTS. Consequently, the number of subjects who participated in each group was smaller than originally planned; however, the subject numbers are consistent with other published literature. For example, Boe et al. [10] found differences in MUNEs when they compared data from 10 healthy subjects to 9 patients with amyotrophic lateral sclerosis (ALS). In the present study, although the age and sex distributions were not significantly different among the groups, ideally subjects would have been matched by age and gender. The small number of subjects recruited prevented matching. Nonetheless, the sample in this study revealed significant group differences in many of the measures studied.

The questionnaire data revealed that there were similar symptom severity scores between the severe CTS group and mild CTS group, and that both groups differed from the control group. The severe CTS group had significantly lower functional scores compared to the healthy control group; however the mild CTS group was not significantly different from either the severe CTS group or the healthy control group. This result is not surprising since sensory loss is normally experienced before motor loss in CTS and as such, the sensory losses experienced in subjects with mild CTS would be similar to those sustained by individuals with severe CTS. Despite the fact that individuals with mild CTS showed no nerve conduction study based evidence of motor loss, the functional implications of their sensory loss explains why their functional scores were not different from the individuals with severe CTS. The sensory and functional scores reported in the current study are within one standard deviation, of the mean values of those reported by Levine et al [18] in patients with CTS who were to undergo surgical repair (Symptom severity: 3.4 ± 0.67; Functional scores: 3.0 ± 0.93).

### Evidence of collateral sprouting detected using DQEMG

The shape characteristics of individual MUPs provide insight into the underlying pathophysiology of neuromuscular disease [5,6]. For example, in individuals with neuropathy, the classic EMG findings are that MUPs with increased duration and amplitudes indicate that collateral reinnervation is occurring or has occurred [5]. In these cases, the complexity of the waveform, as measured by the number of turns and/or phases may either be normal or increased [5]. In the early stages of collateral sprouting, MUP duration and complexity may be increased, whereas in later stages complexity normalizes and amplitude and duration may be unchanged or larger than normal. Stability measurements can also provide useful information regarding what is occurring at the neuromuscular junction, and thus allow inferences about the state of the MUP. Ajiggle measures the amount of shape variation across the selected ensemble of MUP accelerations. Similarly, shimmerCov measures the variation across an ensemble of MUPs. Large values of Ajiggle or shimmerCov may suggest neuromuscular transmission irregularities [11] and can be indicative of early collateral sprouting. Fibre count represents the number of muscle fibres in close proximity to the electrode [11] and, similar to Ajiggle and shimmerCov, increases in fibre count are indicative of collateral sprouting. The results of the current study failed to find significant differences between the groups for any stability measures. The lack of significance may be due to the lack of sensitivity of the stability measures used, or perhaps the three groups had stable neuromuscular transmission. Since all of our subjects with CTS had experienced symptoms for at least three months, signs of early collateral sprouting may have been missed [5]. It should be noted that Ajiggle and ShimmerCov tended to increase with the severity of CTS (Table 4) which might indicate that this study was underpowered in its ability to detect differences in MUP stability in this population.

MUP peak-to-peak amplitude is representative of motor unit size [6]. As such, the larger MUP amplitude in the severe CTS group as compared to the mild CTS and control groups suggests that larger motor units were active during EMG signal detection which in turn may suggest that collateral sprouting may have occurred at some point prior to the study. Similar differences in MUP peak-to-peak amplitude were identified in patients with amyotrophic lateral sclerosis (ALS) using a constant 10% MVC contraction protocol and DQEMG [10]. However, in contrast to Boe et al. [10], we used a constant-intensity protocol so that the three test groups activated a similar number of motor units during EMG signal detection. The intensity of the contraction signifies the aggregate number of MUPs per second (pps) seen in the EMG interference pattern and this was not different among the groups. The constant-intensity protocol required the severe CTS group to contract at a higher percentage of their MVC (close to 40%) during EMG data collection than did the control or mild CTS groups (between 10 and 15% MVC). This resulted in the recruitment of larger motor units [18,19] and is consistent with motor unit loss. This difference in contraction levels between the severe CTS group and the other groups was not surprising since individuals with severe CTS by definition had motor axonal loss [20] and thus, in order to generate an EMG interference pattern of a set level of intensity (i.e. recruit and sufficiently activate a sufficiently large set of motor units) a contraction at a higher level of %MVC relative to their pre-disease state would be required.

In order to investigate the impact of the large differences in contraction intensity between the study groups on the resultant MUP amplitudes and durations recorded from the APB, we recruited an additional sample (n = 5) of healthy individuals and had them undergo the EMG data collection procedures previously described while contracting between 10 and 15% MVC and again while contracting at 40% MVC. The DQEMG results indicated that, although the MUP amplitudes tended to be larger for the higher contraction levels, based on one-way ANOVA results, there was no significant difference in the MUP amplitudes between the two contraction levels (F = 2.45; p = 0.156; See Table 5), which were both substantially lower than the amplitudes seen in the severe CTS group in our study. There was also no difference in MUP duration between the contraction levels (F = 0.00; p = 0.96; See Table 5), which again were much smaller than those seen in the severe CTS group. There were large differences in the contraction intensity between the contraction levels (10-15% MVC: 68 pps; 40% MVC: 82 pps) suggesting that more (and therefore larger) MUs were recruited for the higher level contraction.

**Table 5:** Impact of contraction level on MUP amplitude in a new sample of healthy subjects.

Target Contraction level (%MVC)	Actual Contraction Level (%MVC)	Intensity (PPS)	MUP peak to peak amplitude (uV)	MUP duration (ms)
**10-15%**	12.5 (12.5-14.6)	68 (40-95)	363.6 (232.5-466.3)	6.52 (5.33-8.12)
**40**	42 (38.9-44.7)	82 (73-137)	474.3 (277.4-522.8)	6.30 (5.24-8.19)

**Values presented are medians and ranges**. The differences in MUP amplitude between the two contraction levels were not statistically significant (F-2.45, p = 0.156). * MVC denotes maximal voluntary contraction, PPS denotes pulses per second.

Both MUP and SMUP duration is thought to be influenced by axonal injury, and have been examined previously [10], however MUP durations are also heavily dependent on the distance of the active motor unit to the recording electrode [5]. In the current study, the severe CTS group had significantly longer MUP durations as compared to the mild CTS and control groups. The long MUP durations of the severe CTS group relative to the mild and control groups again suggests that the severe group was undergoing or had undergone collateral reinnervation [5].

The results of the current study offer no evidence that MUPs detected from severe CTS patients have more complexity than those detected from subjects with no motor neuropathy. This might have been related to the high variability inherent in the MUP phase measures [21-23] or again due to a lack of statistical power resulting from the small sample size recruited, since there was a tendency for the severe CTS group to have more phases and turns in their MUP waveforms (Table 3). Other researchers have found low reliably in determining MUP onset and end markers as compared to the high reliability found in determining the peaks [19,21,24]. Calder et al. [19] recently concluded that MUP duration (ICC: -0.29) and the number of phases in the MUP (ICC: -0.69) had poor within-subject reliability. Also using DQEMG, Boe et al. [10] failed to find a difference in complexity between healthy individuals and those with ALS. The number of phases in MUP templates may not be sensitive enough to be used in the study of neuromuscular pathology.

Although MUP morphological characteristics offer insight into the size of the active motor units within a muscle, they are influenced by limitations of the needle electrode used to detect them [22]. Estimating motor unit size and shape using surface EMG electrodes is thought to be a more robust representation, since there is a greater number of muscle fibers per motor unit equally contributing to the surface EMG signal and therefore to the SMUP template [23], and because the relative distance from the active muscle fibers to the detection electrode is essentially the same for all MUs. Despite the absence of significant differences in SMUP morphology among the groups, the trends in SMUP morphology among the groups were similar in pattern to the group differences seen in the MUP morphology measures. This finding is particularly obvious in the SMUP amplitude and area data presented in Table 3. The lack of statistical significance seen in the SMUP parameters may be attributed to the large within-group variability and the small sample size.

Overall, DQEMG appears sensitive enough to determine differences in MUP amplitudes between groups of individuals with and without motor nerve impairment associated with CTS, but in the current study there were no significant differences in measures of MUP stability. The differences in MUP morphology without differences in MUP stability may reflect that collateral sprouting occurred more than three months prior to subjects participating in this study, such that orphaned muscle fibres had been reinnervated and collateral sprouts had matured. In any event, MUP stability measures appear to be of less value in this population.

### Evidence of Motor axon loss detected using DQEMG

MUNEs provide information about the number of functioning motor axons in a given motor unit pool [25-27]. This information is useful when evaluating the extent of motor unit loss associated with motor neuron disease or peripheral neuropathy and when assessing the course and outcome of treatment for these disorders. Using constant %MVC protocols and DQEMG, has been found to be a valid, reliable and practical tool for obtaining MUNEs [8]. However, it has been demonstrated that as the level of contraction used increases the MUNE values decrease [28]. Boe et al. using a 7%MVC contraction level on average have determined normative MUNE values for the APB muscle using SMUP negative-peak amplitude (269 +/- 104) [8]. The median MUNE value of the healthy group in the current study, for which the constant-intensity based protocol resulted in a 10%MVC contraction on average, falls within one standard deviation of Boe et al's reported norm for this muscle. The MUNE values for the mild and severe CTS groups are biased to low values because of the higher level of %MVC produced during EMG signal detection and are not anatomically accurate. Nonetheless, they are valid indicators of motor unit loss when compared to the MUNE values of the control group obtained using the same constant-intensity protocol.

In this study there were significant differences in the all the CMAP amplitudes and the MUNEs between the severe CTS and healthy groups as well as the mild CTS and healthy groups. This result occurred despite the mild CTS group being screened before the study to ensure that they had no clinical evidence of motor involvement [29]. The lack of significant difference found between the mild CTS group and the severe CTS group suggests that at least some individuals in the mild CTS group may have had axonal loss. It is possible, therefore, that MUNEs may provide a more sensitive way to detect motor nerve impairment that is not yet severe enough to be detected using traditional nerve conduction studies. This should be investigated in future studies. The fact that the group with mild CTS did not show evidence of collateral sprouting (increased MUP amplitude and duration relative to the control group) despite having lower MUNEs might indicate that they are at a different stage of the disease process than the severe CTS group.

Unlike other neuropathic conditions such as ALS, where the neuropathy is known to be degenerative in nature, nerve compression injuries can cause both demyelination and axonal loss, both of which can affect the shape characteristics of a CMAP, making it difficult to determine which pathology is most prevalent. Furthermore, it is possible that a portion of the drop in MUNE values is due to reduction in CMAP size due to temporal dispersion of contributing potentials due to conduction slowing which is not accounted for when the mean SMUP is calculated using SMUPs extracted from EMG signal detected during voluntary contractions. Inclusion of a stimulation based MUNE technique might have been informative, but unfortunately was not considered in the design of this experiment. Despite uncertainty in the underlying cause of reduced CMAP size, the consistent trend to increased mean SMUP size across the healthy, mild CTS and severe CTS groups suggest that the amplitude-based MUNE measures are sensitive to differences in the number of healthy or functioning motor units between groups of individuals with and without a given disorder. In this case the severe CTS group (i.e. those with evidence of motor involvement), had lower MUNEs than the mild CTS and control groups.

### Limitations

Sensory, motor, and combined nerve conduction studies were used to stratify individuals by severity of CTS such that we had one experimental group with evidence of motor involvement (severe CTS), one group with sensory involvement but no motor involvement (mild CTS), and a control group. Although the specificity of nerve conduction studies is high, the sensitivities of the different tests is quite variable [3]. The literature suggests that the sensitivities of the motor and mixed nerve conduction studies are lower than those of sensory nerve conduction studies [3,30]. Jablecki et al. [3] reported that the pooled sensitivity (0.85) of the comparison of the median and ulnar sensory conduction between the wrist and the fourth digit proved to be the most sensitive diagnostic test [3]. By contrast, comparisons of median and ulnar mixed nerve conduction between the wrist and palm and motor conduction studies of median nerve across the wrist were reported to have lower pooled sensitivities (0.71 and 0.63 respectively) [3]. It is therefore possible, that our stratification based on symptoms and nerve conduction study results may not have been accurate in all subjects. In particular, in the current study the CMAP morphological features were not significantly different between the mild and severe CTS groups despite the fact that subjects were carefully screened according to the standard guidelines [4].Individuals with mild CTS in the current study may, in fact, have had motor deficits that went undetected based on our criteria. Assessment of abnormal spontaneous activity would have been helpful to rule out motor nerve involvement in our subjects with mild CTS.

## Conclusions

CTS was used as a convenient model to determine the feasibility of using a constant-intensity contraction protocol with DQEMG to detect the presence of motor neuropathy since nerve conduction studies can suggest whether or not individuals with CTS have motor involvement. Despite the different levels of %MVC across the study groups elicited by the constant-intensity protocol the MUPs with significantly larger amplitudes and longer durations in individuals with severe CTS suggest motor unit loss and that orphaned muscle fibers in the participants with severe CTS had undergone collateral reinnervation, however significant changes in MUP stability were not detected using DQEMG. Based on the current findings it appears that quantitative EMG may be a sensitive measure to detect MUP morphological changes in individuals with compressive neuropathy but not necessarily changes in MUP complexity or stability. A much larger study would be required in order to determine the sensitivity and specificity of this approach.

The MUNE results suggest that individuals with severe CTS experience a loss in the number of functioning motor units. The lower MUNEs found in the mild CTS group as compared to the healthy control group suggest that traditional nerve conduction studies may not be as sensitive to subtle motor impairments that may result from early demyelination as are MUNEs and MUP morphological feature values obtained using DQEMG.

## Abbreviations

CTS: carpal tunnel syndrome; ABP: Abductor Pollicis Brevis; DQEMG: decomposition-based quantitative electromyography; EMG: electromyographyl; RMS: root mean square; MU: motor unit; MUP: needle-detected motor unit potential; MUPT: motor unit potential train; MUNE: motor unit number estimate; MVC: maximal voluntary contraction; SMUP: surface-detected motor unit potential; CMAP: compound muscle action potential; CNAP: compound nerve action potential; ALS: amyotrophic lateral sclerosis; SNAP: sensory nerve action potential; RMSMVC: RMS value of the MVC.

## Competing interests

The authors declare that they have no competing interests.

## Authors' contributions

JN and AHW carried out the recruitment and testing of participants, acquisition of data, analysis and interpretation of data. JN drafted the manuscript. MF aided in recruitment of participants as well as analysis and interpretation of data. LM and DWS conceptualized the research question and study design, and provided guidance in terms of data acquisition, analysis and interpretation. LM was the senior researcher and principal investigator of the research study. All authors read and approved the final manuscript.
